# Exophytic Adiposis Dolorosa (Dercum’s Disease) of the Thigh: A Case Report

**DOI:** 10.7759/cureus.7282

**Published:** 2020-03-15

**Authors:** Jude L Opoku-Agyeman, Lauren Coffua, Jamee Simone, Terrance Hanley, Amir Behnam

**Affiliations:** 1 Plastic Surgery, Philadelphia College of Osteopathic Medicine, Philadelphia, USA; 2 Osteopathic Medicine, Philadelphia College of Osteopathic Medicine, Philadelphia, USA; 3 Surgery, Reading Hospital, Wyomissing, USA; 4 Plastic Surgery, Reading Hospital, Wyomissing, USA

**Keywords:** adiposis dolorosa, dercum's diseases, thigh mass, dermolipectomy

## Abstract

Adiposis dolorosa or Dercum’s disease is a rare lipomatous disorder characterized by painful lipomas. In this article, we report a case of rather large exophytic adiposis dolorosa causing difficulties with ambulation, and our surgical management of the disorder. To our knowledge, this is the first reported case of a large exophytic adiposis dolorosa of the upper medial thigh causing problems with mobility. This is also the first reported case of the use of a delayed split-thickness skin graft (STSG) after interval use of wound vacuum-assisted closure (VAC) following dermolipectomy. A 77-year-old female presented with a chronic mass on the medial aspect of her right thigh for over 40-50 years. She had noticed a recent rapid increase in size, causing some discomfort and interference with mobility and activities of daily living. The patient underwent an MRI with finding consistent with adiposis dolorosa. She underwent dermolipectomy and reconstruction of the resulting defect with a combination of partial primary closure, wound VAC, and delayed closure using STSG. Dermolipectomy with interval application of a wound VAC combined with delayed reconstruction with STSG is a feasible option for patients with large lesions of the extremity that causes difficulty with mobility and activities of daily living.

## Introduction

Adiposis dolorosa or Dercum’s disease is an extremely rare disease with characteristic painful lipomas. It is classified under rare diseases by the National Organization for Rare Disorder (NORD) [1]. This entity was first described by Francis Xavier Dercum in 1862 [2]. Dercum’s disease is a predominantly female disease occurring between the ages of 35-50 years [3]. Patients are typically overweight or obese, and the disease causes significant pain in the adipose tissue. The pain associated with it has been described as being chronic in nature. The most commonly affected area are the extremities, trunk, pelvis area, and buttocks [3]. Management of this disorder is very difficult, as there is currently no permanent therapy for the condition. The management has mainly been focused on pain control and has ranged from medical management to surgical management. The different options of surgical management include liposuction and dermolipectomy [4-9].

Most of the reported cases describe patients with diffuse or localized adiposis dolorosa. We report the surgical management of a patient with a large exophytic adiposis dolorosa of the medial thigh whose main complaint was pain and difficulty with mobility and activities of daily life. To our knowledge, this is the first reported case of a large exophytic adiposis dolorosa of the medial thigh causing problems with mobility, and the first reported case of the use of a delayed split-thickness skin graft (STSG) after interval use of wound vacuum-assisted closure (VAC) following dermolipectomy.

## Case presentation

A 77-year-old female was seen in our plastic surgery clinic for evaluation of a right medial thigh mass. The patient was referred to us by a general surgeon who had recommended resection and required assistance with the reconstruction of the resulting defect. The patient had had the mass for over 40-50 years. The mass had rapidly enlarged over the previous six months and was interfering with walking, sitting, and the ability to wear clothing. She reported some localized discomfort and pain but denied any paresthesia or signs and symptoms of claudication. She had not sought any intervention for the mass in the past.

**Figure 1:**
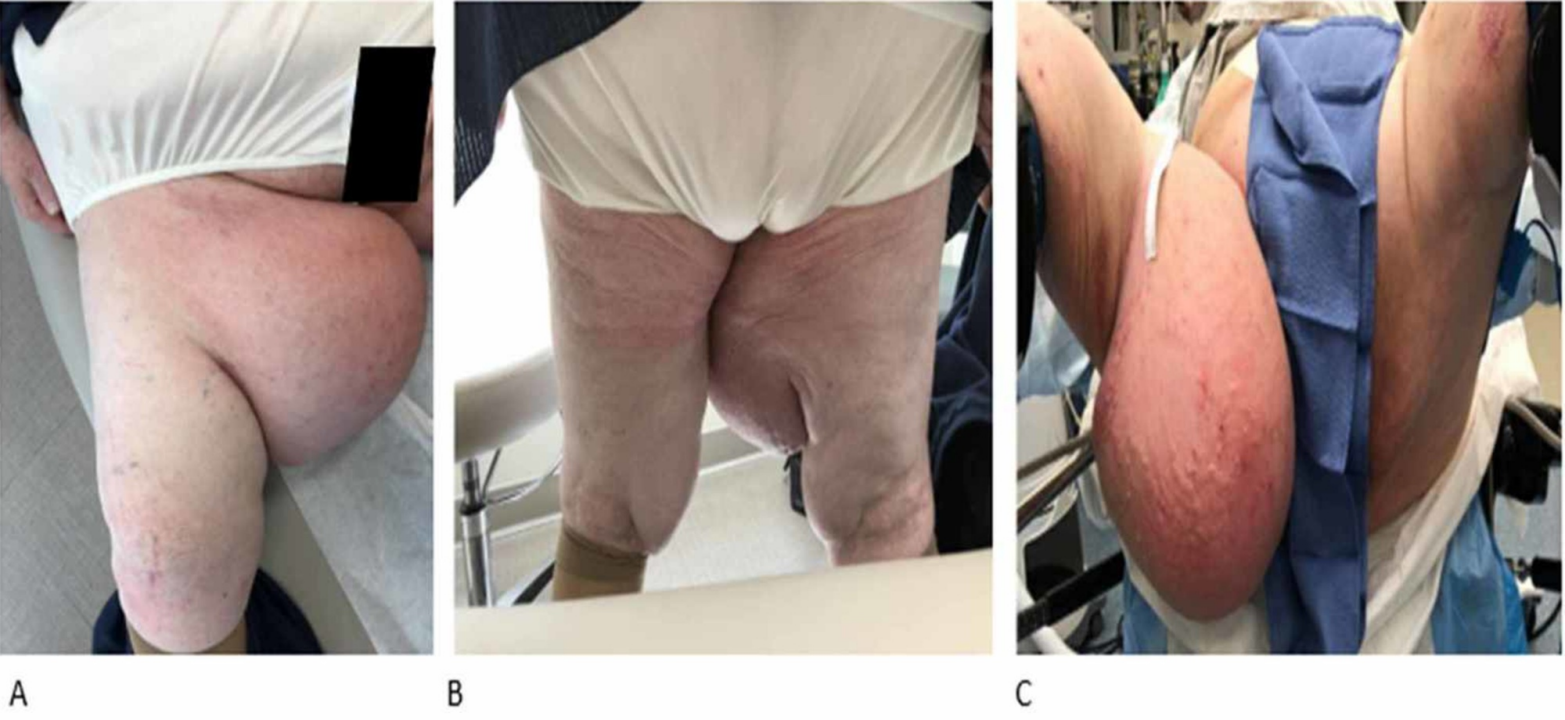
Large adiposis dolorosa on medial aspect of right thigh A: patient sitting; B: patient standing; C: patient just before surgical resection

**Figure 2:**
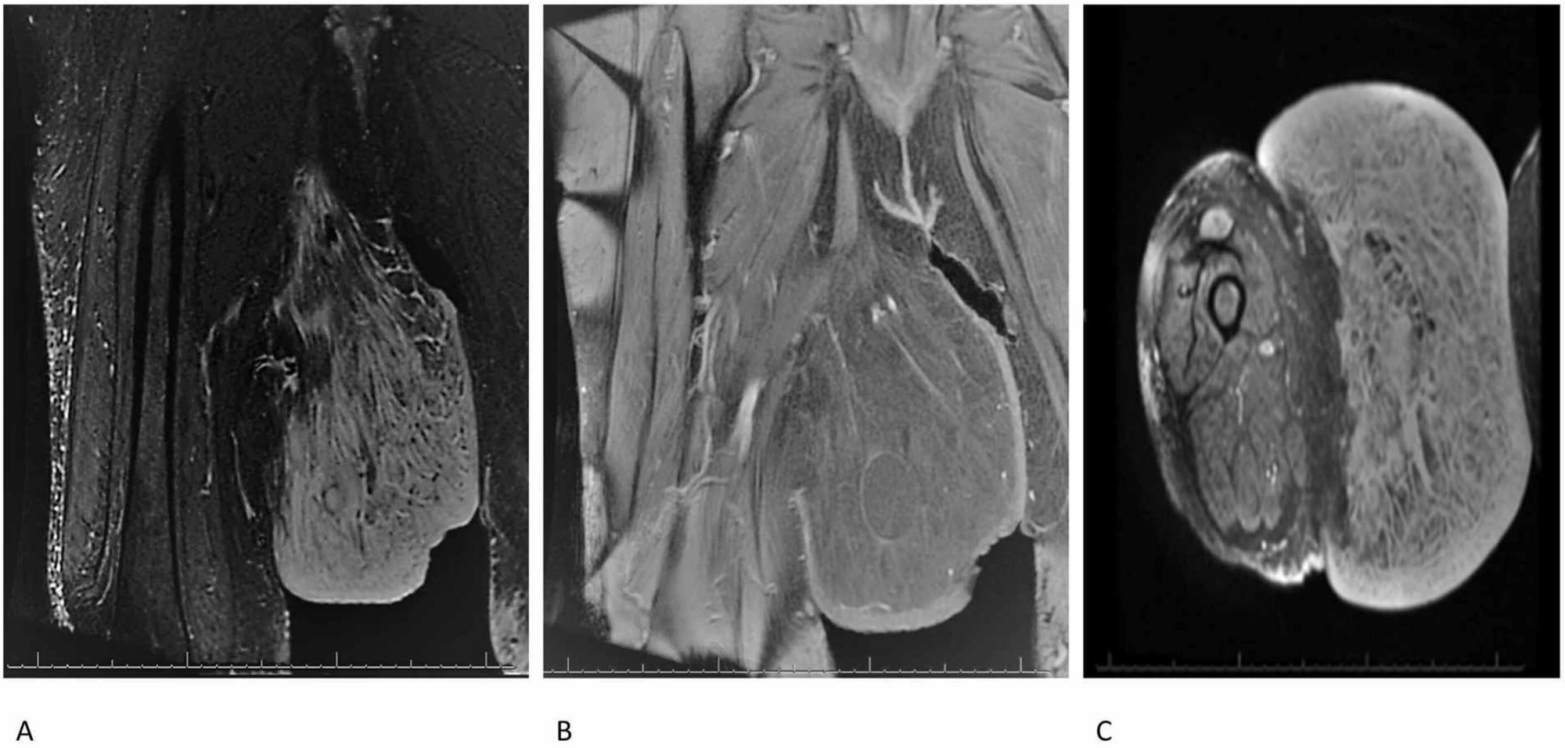
Preoperative magnetic resonance images of the lesion A and B: coronal plane; C: axial plane

Her past medical history was significant for hypertension and obesity. Her physical examination revealed a very large mass protruding from the right medial thigh, measuring more than 40 cm in its greatest dimension (Figure 1). The color and texture of the overlying skin were normal and without ulcerations. The contralateral thigh was normal for her body habitus. Laboratory studies, including complete blood count and basic metabolic panel, were within normal limits. Based on the size of the mass and physical characteristics, our differential diagnosis included a large lipoma versus a sarcoma. An MRI with and without gadolinium had been obtained by the general surgeon and revealed a very large superficial exophytic mass in the right medial thigh with characteristics most suggestive of adiposis dolorosa (Figure 2).

**Figure 3:**
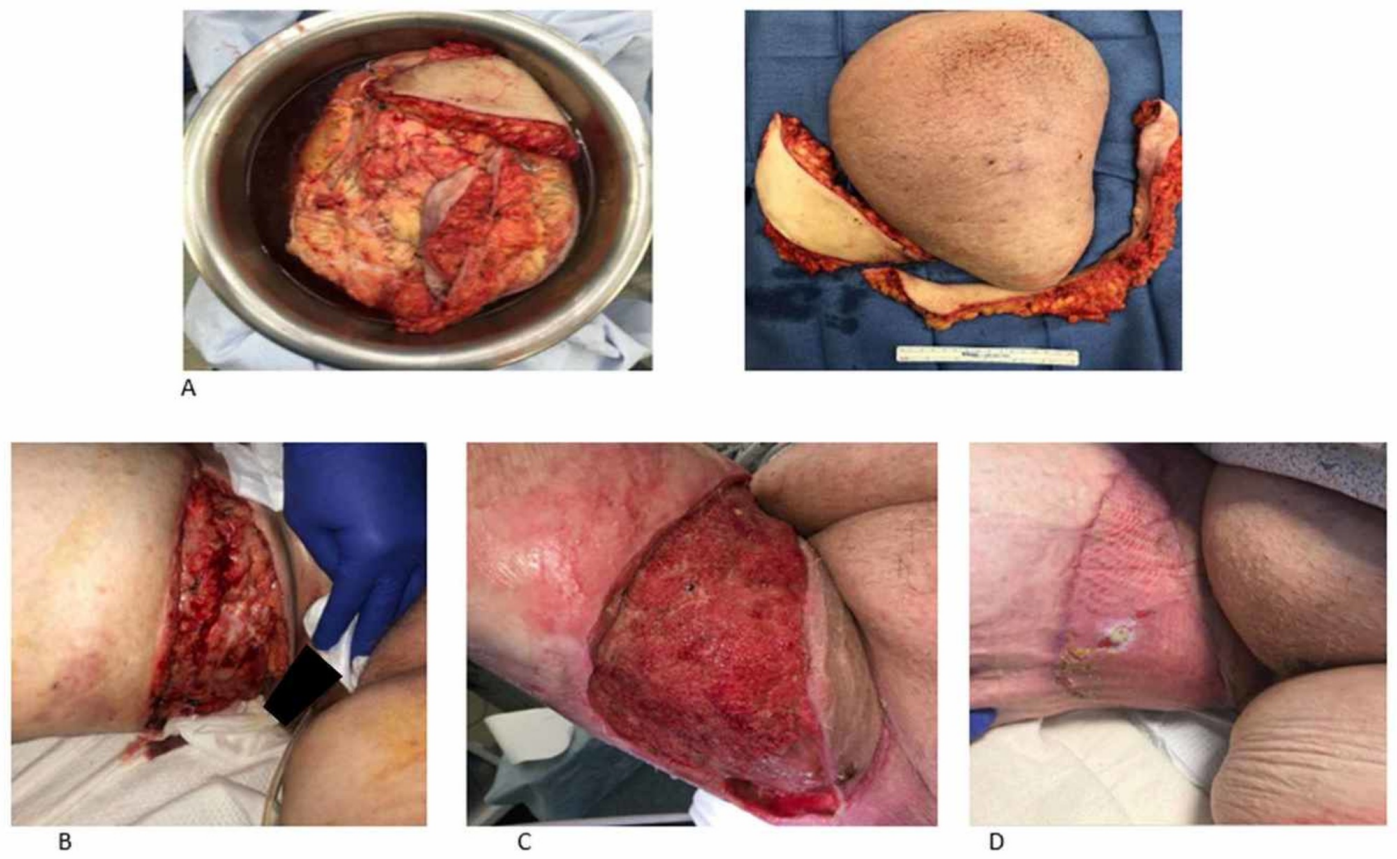
Intraoperative and postoperative images A: resected tissue; B: large defect after resection of the lesion; C: wound bed 18 days after VAC therapy with healthy granulation tissue; D: well-taken STSG seven weeks after the initial surgery VAC: vacuum-assisted closure; STSG: split-thickness skin graft

The patient underwent a dermolipectomy and delayed reconstruction. The procedure involved resection of the skin and the subcutaneous tissue (dermolipectomy). The mass had not invaded the muscles or the fascia. The patient was noticed to have large fatty tissue within the mass. The resected tissues were sent for pathological analysis (Figure 3A). The resulting defect was about 50 x 15 x 1 cm and could not be closed primarily (Figure 3B). Part of the wound was closed primarily, and a wound VAC was placed on the remainder to help prepare the wound bed for delayed closure with STSG. The patient underwent multiple wound VAC changes and the wound was deemed ready for skin grafting when there was evidence of healthy granulation tissue in the wound bed (Figure 3C). She underwent placement of STSG and placement of wound VAC to secure the graft 18 days after the initial surgery. The wound VAC was eventually taken down one week later, and there was excellent take of the STSG. Seven weeks after the procedure, the skin graft was healthy and there was only a negligible amount of contour deformity (Figure 3D). The patient also reported no discomfort and was able to ambulate, sit, and wear clothing with no difficulty. The postoperative pathological report revealed edematous fibro-adipose tissue and chronic inflammation, consistent with adiposis dolorosa. Her appearance has improved, and she is able to walk, sit, and wear underwear without any difficulty. There is currently no physical evidence of recurrence.

## Discussion

Adiposis dolorosa or Dercum’s disease is a rare and predominantly female disease. It is characterized by the presence of painful subcutaneous adipose tissue in overweight or obese individuals. The current management of this condition includes medical as well as surgical management to control the symptoms, mostly pain. Medical management is aimed at controlling the pain associated with the condition. Some of the modalities include corticosteroids, lidocaine infusion, and methotrexate [10]. These pharmacological options do not affect the mass of the adiposis dolorosa, especially in cases where the mass is causing difficulties with mobility.

Surgical management including liposuction and dermolipectomy may be of use in the subset of patients who do not benefit from pharmacotherapy or have limitations in their mobility [9,11]. Our patient presented with a large exophytic mass interfering with her daily activities. The concerns of the patient could not have been ameliorated with medical management alone. Liposuction would not have been effective as the mass was not diffuse, but rather large and exophytic. Moreover, liposuction has not been found to be effective in long-standing disease due to fibrosis [11,12]. Dermolipectomy has been found to be effective but is associated with pain recurrence [7-9,13,14]. Since our patient had minimal discomfort and pain, our main aim was surgical resection to rectify her mobility issues.

Dermolipectomy as a treatment for this condition has not been extensively studied, and most of the studies are limited to case reports and case series. Some of the reported complications of dermolipectomy include pain recurrence, recurrence of the collection, and lymph fistula [7,13-15]. Other reconstructive modalities could have been considered, including primary closure or immediate skin grafting. We opted against primary closure due to the size of the defect. Immediate closure with STSG was not considered ideal because the wound bed was mostly composed of fatty tissue and the graft take would more than likely have been poor. In reconstructing this defect, healthy granulation tissue needed to be recruited to the wound bed to allow the STSG to have a good take. Our patient had a very good recovery from the procedure, and there are no signs of recurrence or discomfort at the surgical site currently, seven weeks after the procedure. Our study is limited by the short follow-up time. She will be followed up in the long term, if possible, to monitor for recurrence and developments of complications relating to the surgery.

## Conclusions

Adiposis dolorosa is a very rare condition. In this article, we reported the surgical management of a single case at our institution. Management is mostly focused on pain control and can be achieved through observation, medications, or surgery. Dermolipectomy is very effective for large lesions of the extremities to improve mobility. However, these resections can leave large defects unamenable to primary closure alone. Resection with interval application of wound VAC combined with delayed closure with STSG is a feasible alternative.
